# Screening and brief intervention for obesity in primary care: a parallel, two-arm, randomised trial

**DOI:** 10.1016/S0140-6736(16)31893-1

**Published:** 2016-11-19

**Authors:** Paul Aveyard, Amanda Lewis, Sarah Tearne, Kathryn Hood, Anna Christian-Brown, Peymane Adab, Rachna Begh, Kate Jolly, Amanda Daley, Amanda Farley, Deborah Lycett, Alecia Nickless, Ly-Mee Yu, Lise Retat, Laura Webber, Laura Pimpin, Susan A Jebb

**Affiliations:** a Nuffield Department of Primary Care Health Sciences, University of Oxford, Radcliffe Observatory Quarter, Oxford, UK; b School of Social and Community Medicine, University of Bristol, Bristol, UK; c Institute of Applied Health Research, University of Birmingham, Birmingham, UK; d Faculty of Health and Life Sciences, Coventry University, Coventry, UK; e UK Health Forum, London, UK

## Abstract

**Background:**

Obesity is a common cause of non-communicable disease. Guidelines recommend that physicians screen and offer brief advice to motivate weight loss through referral to behavioural weight loss programmes. However, physicians rarely intervene and no trials have been done on the subject. We did this trial to establish whether physician brief intervention is acceptable and effective for reducing bodyweight in patients with obesity.

**Methods:**

In this parallel, two-arm, randomised trial, patients who consulted 137 primary care physicians in England were screened for obesity. Individuals could be enrolled if they were aged at least 18 years, had a body-mass index of at least 30 kg/m^2^ (or at least 25 kg/m^2^ if of Asian ethnicity), and had a raised body fat percentage. At the end of the consultation, the physician randomly assigned participants (1:1) to one of two 30 s interventions. Randomisation was done via preprepared randomisation cards labelled with a code representing the allocation, which were placed in opaque sealed envelopes and given to physicians to open at the time of treatment assignment. In the active intervention, the physician offered referral to a weight management group (12 sessions of 1 h each, once per week) and, if the referral was accepted, the physician ensured the patient made an appointment and offered follow-up. In the control intervention, the physician advised the patient that their health would benefit from weight loss. The primary outcome was weight change at 12 months in the intention-to-treat population, which was assessed blinded to treatment allocation. We also assessed asked patients' about their feelings on discussing their weight when they have visited their general practitioner for other reasons. Given the nature of the intervention, we did not anticipate any adverse events in the usual sense, so safety outcomes were not assessed. This trial is registered with the ISRCTN Registry, number ISRCTN26563137.

**Findings:**

Between June 4, 2013, and Dec 23, 2014, we screened 8403 patients, of whom 2728 (32%) were obese. Of these obese patients, 2256 (83%) agreed to participate and 1882 were eligible, enrolled, and included in the intention-to-treat analysis, with 940 individuals in the support group and 942 individuals in the advice group. 722 (77%) individuals assigned to the support intervention agreed to attend the weight management group and 379 (40%) of these individuals attended, compared with 82 (9%) participants who were allocated the advice intervention. In the entire study population, mean weight change at 12 months was 2·43 kg with the support intervention and 1·04 kg with the advice intervention, giving an adjusted difference of 1·43 kg (95% CI 0·89–1·97). The reactions of the patients to the general practitioners' brief interventions did not differ significantly between the study groups in terms of appropriateness (adjusted odds ratio 0·89, 95% CI 0·75–1·07, p=0·21) or helpfulness (1·05, 0·89–1·26, p=0·54); overall, four (<1%) patients thought their intervention was inappropriate and unhelpful and 1530 (81%) patients thought it was appropriate and helpful.

**Interpretation:**

A behaviourally-informed, very brief, physician-delivered opportunistic intervention is acceptable to patients and an effective way to reduce population mean weight.

**Funding:**

The UK National Prevention Research Initiative.

## Introduction

Internationally, guidelines recommend that physicians screen for and opportunistically encourage patients to lose weight.^1^, ^2^ However, patient surveys and recordings of consultations suggest that such guidelines are not widely implemented.^3^, ^4^ Physicians report several barriers to action, including insufficient time and knowledge, belief that intervention would be ineffective, and fear of causing offence.^5^, ^6^, ^7^, ^8^ However, patients seem to be open to receiving advice from their doctors.^9^

No randomised trials have investigated whether advice from physicians leads to weight loss in their patients. Systematic reviews of data from randomised trials show strong evidence that brief physician intervention is effective for smoking cessation and some evidence that it is effective at reducing problem drinking,^10^, ^11^ suggesting that brief opportunistic interventions on behavioural risk factors can be effective. Cross-sectional data show that people who are attempting to lose weight are more likely to report having received advice from a physician than are people who aren't trying to lose weight.^12^, ^13^, ^14^ However, recordings of consultations show that it is the patient, not the doctor, who instigates half of these discussions,^4^ which could explain the association; patients motivated to lose weight seek help from their physician. We therefore did a trial to test the effectiveness of physicians screening for and opportunistically intervening on obesity.

**Research in context**

**Evidence before this study**

A US Preventive Services Task Force systematic review searched for studies of screening and opportunistic intervention on obesity up to September, 2010, and found no studies. On Sept 23, 2016, we updated this search in MEDLINE, PsycINFO, and the Cochrane Central Register of Controlled Trials, restricted to articles published in English. We found no trials. International guidelines recommend that physicians screen for obesity and offer referral to effective weight management programmes. This recommendation is based on the results of randomised trials showing such programmes to be effective in people seeking treatment, but no trials have examined whether screening and opportunistic intervention in people not seeking support are effective or acceptable. Physicians rarely intervene opportunistically.

**Added value of this study**

To our knowledge, this is the first trial of screening and opportunistic intervention on obesity in the world. We enrolled consecutive patients presenting to a primary care physician who were obese. We trained physicians to offer referral to an effective weight management programme, ensure that the patient left with an appointment, and offer follow-up, and to do so within 30 s. In the control intervention, the physician advised the patient that their health would benefit from weight loss. We showed that such brief interventions were highly acceptable, with most patients finding opportunistic intervention appropriate and helpful and very few finding it inappropriate and unhelpful. Moreover, the intervention group lost more than the control group at 12 months. Most people tried to lose weight during the year of follow-up, with only a small difference between groups. The difference in weight loss arose mainly because of the greater uptake of behavioural support for weight loss in the intervention group.

**Implications of all the available evidence**

Evidence suggests that physicians are concerned about offending their patients by discussing weight, but qualitative evidence from patients and this trial in particular shows that they should be less concerned. If physicians act in accordance with the guidelines, patients are likely to welcome the intervention and lose a significant amount of weight. Given that many patients consult their primary care physician at least once a year and most several times a year, this brief intervention has high reach, is practicable, and could reduce population mean weight.

## Methods

### Study design and participants

The full trial protocol has been reported previously^15^ and is available online. The protocol was implemented without changes. This study was a parallel, two-arm, randomised trial of a brief intervention for obesity in primary care, which involved the participation of 137 primary care physicians at 57 practices from across the south of England. The trial was approved by the NHS Research Ethics Service.

Study researchers attended participating primary care practices and asked to weigh, measure, and estimate the body fat of every patient waiting to see a physician by use of a Tanita SC-240MA Body Composition Analyser (Tanita, Amsterdam, Netherlands). We sought informed consent from all patients. To be enrolled, participants needed to be aged at least 18 years, have a body-mass index (BMI) of at least 25 kg/m^2^ if they were of Asian ethnicity^16^ or at least 30 kg/m^2^ if they were of any other ethnic groups, and have a raised body fat percentage (defined in accordance with age and sex).^17^ Participants who declined participation were asked for anonymous data on their demographic characteristics and height and weight.

We excluded women who were pregnant or planning a pregnancy within 12 months, people who had undergone bariatric surgery at any time, people who had completed a weight management programme (pharmacotherapy or behavioural programme) within the past 3 months or were currently enrolled in one, people who were attending the physician to discuss weight, or people who could not speak English. People who consented and were eligible to participate were handed a randomisation envelope to give to the general practitioner (GP), which included an appended record of the patient's height, weight, and BMI. Physicians could exclude people during the consultation, before opening the randomisation envelope (in which case the envelope was reused), if opportunistic intervention on weight was clinically inappropriate (eg, short life expectancy or a history of eating disorder), inappropriate in that consultation (eg, an emotional consultation), or for other exceptional reasons.

### Randomisation and masking

An independent statistician used Stata Software version 12 to produce a randomisation list that was stratified by physician, with random permuted blocks of four. The list was used to prepare randomisation cards, which were placed in opaque sealed envelopes. Neither the researchers nor the physicians enrolling participants were aware of the allocation for each potential participant. Once the physician opened the envelope, the randomisation card contained a two letter code showing the assigned intervention, which was either support or advice. Sealed envelopes provide a faster method of enacting randomisation than do any other methods, so it did not break the flow of the consultation.

Half of the randomisation cards also carried a request to record the consultation about weight, although patients were allowed to opt out or could request deletion afterwards. The sequence for random allocation of recording was prepared in exactly the same way as for allocation to intervention, but was independent of it.

### Procedures

We considered and decided against using a control condition that offered no intervention. Such a control would have compromised our ability to assess feelings about the intervention and compromised blinding. Instead, for the control, we used an intervention that physicians typically use when intervening on behavioural risk factors: advice to change behaviour to benefit health.^6^ Physicians were allowed to personalise this advice on the basis of their patient's medical or family history.

The aims for the active intervention were that it would be effective, acceptable, and could be delivered in less than 30 s, to meet physicians' concerns about implementation. The design of the intervention was informed by evidence that an offer of help to change is more motivating than advice to do so.^18^ We therefore encouraged physicians to offer referral to a weight loss service that has proven effective. Such programmes are widely available to primary-care physicians in England, are usually provided by commercial weight loss companies, and are the recommended first-line intervention for obesity, having been shown to be effective.^19^, ^20^, ^21^ In this trial, these programmes were provided mainly by Slimming World (Alfreton, UK). When patients are referred for free through the NHS, the programme offers 12 sessions consisting of 1 h of behavioural group support, once per week. Second, we drew on the results of a trial for smoking cessation that showed that brief opportunistic interventions encouraging patients to use a behavioural programme have a ten-times higher uptake when the referral is enacted by the system rather than leaving patients to instigate it.^22^ We ensured that patients who agreed to referral left the practice with an appointment. Finally, we drew on evidence that external accountability is an important component of behavioural programmes^23^ and we trained physicians to ask the participant to return in 4 weeks to assess their progress (panel).

We trained physicians with a 90 min online course. The modules covered the rationale of the trial, the medical benefits of weight loss, and the mechanics of running the trial, but mostly consisted of filmed consultations with commentary to help physicians assimilate the skills necessary to deliver both interventions with confidence. The course also trained physicians to handle difficult situations that might arise in consultations and what to do in follow-up consultations.

We assessed fidelity by recording randomly selected consultations (ie, consultations in which the randomisation card included a request to record). After each physician's session, the researcher listened to the recording and assessed whether key aspects of the intervention were delivered as intended. We provided feedback to physicians where necessary to improve fidelity. These recordings were prespecified in the protocol analysis plan.

### Outcomes

The primary outcome was weight change from baseline to 12 months. Secondary outcomes were the proportion of participants who had lost 5% and 10% of their baseline body weight at 12 months and mean change in self-reported weight from baseline to 3 months. The secondary outcomes also included cost per kg, which is presented briefly in the discussion, and cost per kg per m^2^. We will present a fuller economic analysis in a separate paper. We used a checklist to code a convenience sample of the consultation recordings for the presence or absence of key aspects of the control and active interventions. Fidelity in the intervention group was assessed by recording whether or not an offer of referral was made and whether or not there was supporting discussion (for example, encouragement to attend, advice on the superiority of the service over trying alone). Fidelity in the control group was assessed on whether advice was given that linked weight loss to improved health and that no offer of referral to weight management was made. To assess patients perception of the interventions, participants completed two questions on their response to the physician's brief intervention immediately after the consultation. At 3 months, participants were telephoned by researchers blinded to allocation to assess their actions to manage weight. Researchers masked to allocation weighed participants at 12 months using the same Tanita device as at baseline and again assessed participants' actions on weight. These actions were classified as no action, self-help measures (alterations to diet and activity), and more effective than self-help measures (attending a behavioural weight loss programme, taking orlistat, or following a meal-replacement weight loss programme). Finally, we also assessed physicians' thoughts, feelings, and practice about giving opportunistic interventions on weight before and after participating in the trial. These outcomes will be reported separately. Given the nature of the intervention, we did not anticipate any adverse events in the usual sense so safety outcomes were not assessed.

### Statistical analysis

Our sample size was based on the assumption that people lost to follow-up would be imputed as not having changed weight: ie, baseline observation carried forward (BOCF).^24^ Although the assumption is often made that people gain weight over time, this notion seems to be untrue for people who are overweight. The Prospective Studies Collaboration did a meta-analysis of data from 72 000 participants with a BMI more than 30 kg/m^2^ and their results showed modest weight loss, so BOCF seemed a reasonable and conservative approach.^25^ We assumed that we would be able to follow-up 70% of participants, that 30% of the participants would take up referral for weight management when offered, and that in doing so these individuals would lose 3·5 kg at 1 year.^19^ We assumed that 10% of the control group would aim to lose weight and achieve 1·2 kg weight loss.^19^ Assuming an SD for weight change of 6·0 kg,^19^ to achieve 90% power and 5% type I error, we would need to randomly assign 912 people to each group, giving 1824 in total.

The primary analysis of weight change at 12 months was based on the intention-to-treat principle, with BOCF-imputed values where data were missing. We used a linear mixed-effect model to assess weight change at 12 months, adjusting for baseline weight as a fixed effect and physician as a random effect. We also did a prespecified sensitivity analyses that included complete case analysis and several other imputation methods to assess the robustness of the results. We used the same model to analyse weight change at 3 months. For the other secondary outcomes, proportion of patients losing 5% and 10% of baseline weight, we compared between groups with an analogous logistic model.

We summarised the appropriateness and helpfulness data separately and combined them to give the proportion of participants who thought the intervention was both inappropriate and unhelpful or appropriate and helpful to any degree, and we used a mixed-effects ordinal logistic regression model to compare between treatment groups. We analysed participants' actions to manage their weight at 3 months and 12 months, which we categorised as no action, self-help, and effective action, with a similar ordinal logistic model. We present fidelity data descriptively by item.

All statistical analyses were done in accordance with a prespecified analysis plan, which is available from the authors on request. We used Stata Software version 13·1 for all analyses and we defined statistical significance as a p value less than 0·05 (2 sided). This trial is registered with the ISRCTN Registry, number ISRCTN26563137.

### Role of the funding source

The funder of the study had no role in study design, data collection, data analysis, data interpretation, or writing of the report. PAv, AN, and L-MY had full access to all the data in the study and PAv had responsibility for the decision to submit.

## Results

Between June 4, 2013, and Dec 23, 2014, we screened 8403 patients. Of these individuals, 2728 (32%) had a BMI defined as obese with raised body fat percentage and were offered enrolment: 2256 (83%) individuals agreed to participate, of whom 1882 (83%) of these were eligible and enrolled. Researchers excluded 259 (11%) individuals, the main reason being that the patient was already taking action on weight, and physicians excluded 122 (5%) individuals, but mostly did not record the reason. 940 participants were assigned to the support (active) intervention and 942 to the advice (control) intervention. One individual (<1%) assigned to the active intervention and three (<1%) individuals assigned to the control intervention received the intervention that was not assigned because of physician error, but were analysed as allocated (figure). We weighed 1419 (75%) of participants at the 12 month follow-up.

Overall, the mean age of participants was 56·0 years (SD 16·1), 1076 (57·2%) of 1882 were women, and 96 (5%) individuals were from minority ethnic groups. Mean weight was 104·6 kg (SD 15·7) for men and 92·5 kg (15·3) for women, with a mean BMI of 34·9 kg/m^2^ (4·8). Characteristics were balanced between treatment groups (table 1). We found no large differences between the characteristics of people who declined participation or were ineligible and those included, except that women were slightly less likely to be willing to participate (282 [17%] women *vs* 168 [16%] men were unwilling to participate).

Weight loss at 12 months, the primary outcome, was 2·43 kg (SD 6·49) for the support group and 1·04 kg (5·50) for the advice group, giving a difference of 1·43 kg (95% CI 0·89–1·97, p<0·0001). Mean self-reported weight loss at 3 months was 2·91 kg (5·16) for support and 1·18 kg (3·81) for advice, giving a difference of 1·76 kg (1·35–2·17, p<0·0001).

Overall, participants found the interventions both appropriate and helpful, with 1530 (81%) of 1882 individuals describing the intervention as both appropriate and helpful, whereas only four (<1%) individuals found the intervention inappropriate and unhelpful. The ratings that participants gave did not significantly differ between the support intervention and the control intervention (table 2).

As a result of the support intervention, 722 (77%) of 940 participants accepted referral to the weight management programme and 379 (40%) attended an appointment, compared with 82 (9%) participants who were allocated the advice intervention. The odds of a patient taking effective action to manage their weight were significantly higher in the support group than in the advice alone group (table 3).

At 12 months, 238 (25%) of 940 participants in the support group had lost at least 5% of their bodyweight and 117 (12%) had lost at least 10%. These proportions were roughly double those in the control group (131 [14%] of 942 individuals lost 5% of bodyweight and 53 [6%] lost 10% of bodyweight; table 4).

Physician fidelity was reasonably good (appendix). Physicians reported that patients very rarely re-attended for review. Listening to the recordings, these apparent failures are often explained by the direction of the consultation. For example, if on starting to describe the possibility of referral to a weight management service, a patient made it clear that she or he would not want a referral, physicians would not go on to make a direct offer of referral in that case, which was appropriate. In 18 (17%) of 106 recordings, physicians spoke to patients indirectly, using phrases such as “the trial would like to offer you referral”. Recordings of this kind were often marked by hesitancy and we interpret this inability to personally take ownership of the advice as reflecting the physician's nervousness about so directly addressing patients about their weight in the consultation. The last two elements of the brief intervention, asking patients to make an appointment at the weight management service and offering follow-up, were intended only for people who accepted the initial referral: ie the 722 (77%) patients in the support group who accepted referral. Consequently, adherence to these referrals was somewhat higher than the figures suggest.

In our prespecified sensitivity analyses, changing the assumptions about imputed weight for those lost to follow-up did not change the results of the primary analysis (appendix).

## Discussion

A brief, 30 s, opportunistic intervention delivered by trained primary care physicians together with a supportive system meant that roughly 40% of patients attended the weight management group offered and 54% of the patients assigned to the active intervention took effective action to manage their weight compared with 11% of the control participants. Consequently, participants who received advice and support lost 1·43 kg more than did those who received advice. Only four (<1%) people thought the opportunistic interventions were inappropriate and unhelpful and most participants thought the opposite, with no difference in response between treatment groups.

Previous studies on this topic have been cross-sectional,^26^ so the major strength of this study is its prospective randomised design. Importantly, most eligible people participated in the trial and there were no substantial differences in the demographic profile of those recruited and those who declined. However, the routine recruitment of participants and our desire to make the intervention from the physician truly opportunistic limited the information that we could collect at baseline; we did not obtain data on participants' ratings of desire, intention, or confidence to achieve weight loss. The absence of these data limits our ability to examine whether either intervention increased participants' motivation to lose weight, although we excluded people who were already taking action to manage their weight, suggesting that we recruited people with low motivation to lose weight. Follow-up is difficult in weight loss trials, with typical 1 year follow-up of 63%.^27^ In this trial, in which the population were not seeking help to lose weight, follow-up was particularly difficult, but we managed to measure the weights of 1419 (75%) participants at 12 months; imputation with various methods and completer analysis did not change the findings. The study participants were recruited from southern England and the only large conurbation that we recruited from was Bristol. Consequently, most participants were of white ethnicity. Although a range of people from different socioeconomic circumstances were included, the study population was slightly more affluent on average than the population of England as a whole. Whether the results can be generalised to other groups depends on whether the response to the physician's intervention differs by socioeconomic or ethnic group and whether the response to the weight loss programme varies. Existing evidence^20^ suggests that no differences in response seem to exist by socioeconomic or ethnic groups. The results might therefore be applicable to similar populations where physicians are able to refer patients to an effective weight management programme provided at no cost to the patient, as is the case in the UK, Australia, or Germany. In health-care systems in which free treatment is not available, arranging a referral at the patient's own cost might possibly also be effective, although not everyone will be able to afford the weekly fee (about £5 in the UK).

In most consultations, physicians do not discuss weight with patients who are obese.^4^, ^28^ In the control group, physicians provided strong advice that losing weight would benefit participants' health and it is possible that this advice alone might have been effective. Results from a meta-analysis of general population cohorts suggests that people who are obese lose 300 g a year in the course of normal living.^25^ On average, individuals in who only received this advice lost more than 1 kg, which is typical of a population motivated to lose weight.^29^ Other data from our trial also suggest that physician's advice motivated patients and prompted action. In the population screened, more people had attended a weight management group in the 3 months after the intervention than in the 3 months before the GP's advice.

Most people who were obese in this unselected population took action during the year to lose weight. Although the active brief intervention increased the proportion of people taking some action from 75% of to 86% (table 3), it increased the proportion of people who were taking effective action by five times. This five times increase in the use of a routinely available behavioural weight management programme created the difference in weight loss between treatment groups, with individuals who attended a weight loss programme lost substantially more weight than those who did not attend one. These programmes are widely available for primary care and some secondary care physicians to refer patients to in the English NHS. However, available data suggest that such referrals are rare.^30^ The implication for practising physicians is that they should concentrate on directing patients to effective support rather than seeking to boost inherent motivation to take any action at all. For behavioural scientists who are developing future brief interventions, it is notable that this intervention seems to have increased motivation to act by providing an opportunity to do so, rather than just reasons to act. Also notable is the fact that weight loss in this initially unmotivated group who attended the weight loss programme was similar to that seen in people seeking help to lose weight.^19^, ^20^ Intrinsic motivation might not be key to success in well-structured behavioural weight loss programmes.

Guidelines recommend that physicians offer brief opportunistic interventions to patients who are obese, and, to our knowledge, this trial is the first to directly support these recommendations.^1^, ^2^ The recommendations are based on evidence from trials that behavioural weight management programmes are effective.^19^, ^20^, ^21^ However, these trials have involved people actively seeking help from a physician to lose weight. No previous trial has approached people opportunistically at visits in which patients were consulting about problems unrelated to their weight and offered treatment, as physicians did here (panel).

Initially, physicians participating in this trial reported that they were nervous about offering unprompted interventions on weight and that they had often become embroiled in long and fruitless conversations on weight in the past. The results from this trial should provide strong reassurance and a practical way forward. Additionally, the costs of this intervention were modest. GPs reported that patients returned for follow-up rarely or never, so the intervention takes 30 s of the physician's time, costing roughly £1·45, and the 12 week behavioural support programme that the physicians referred patients to cost £50. In this trial, research assistants booked patients into the weight management programme. This task—ie, opening the website of the provider and finding a convenient day and time and transferring the details to a voucher—could easily be completed by the physician's administrative staff, such as receptionists. This would cost about £0·76 for the 2 min it takes to complete. Given that 40% of patients in the active intervention group attended the programme, each brief intervention cost the NHS about £22, giving a cost per kg lost at 12 months of £16. This cost per kg is many times less than that of other available interventions for obesity, such as prescription of pharmacotherapy. Although prescription could add to the effectiveness of brief interventions, this low cost per kg makes the case that brief interventions could reasonably be viewed as the first option in physicians' time and effort spent on obesity. A fuller cost-effectiveness analysis will be published elsewhere. In the UK and USA, more than 80% of patients attend their primary care physician every year, making five and three visits per year, respectively.^31^, ^32^, ^33^, ^34^ In a post-hoc analysis, we used an established microsimulation model to examine the cumulative health effect of physicians making a brief intervention on just one of these visits each year.^35^ According to our simulation, by 2035, because of the falling prevalence of obesity, the annual incidence of coronary heart disease, hypertension, and diabetes could be 22%, 23%, and 17% lower than predicted in the base case model in the UK and 9%, 21%, and 20% lower in the USA. This frequency of brief interventions would take a primary care physician about 2·5 h per year in the UK and 4 h per year in the USA to deliver, but would probably save much more time in reduced consultations for these chronic conditions.

In conclusion, a brief opportunistic intervention by physicians to motivate weight loss in unselected patients who are obese was highly acceptable to patients. When combined with supportive systems, the intervention led to overall population weight loss.

For the **trial protocol** see https://ora.ox.ac.uk/objects/uuid:8ae2ebb0-07d7-4671-81b8-06c9d34461fa

## Figures and Tables

**Figure:**
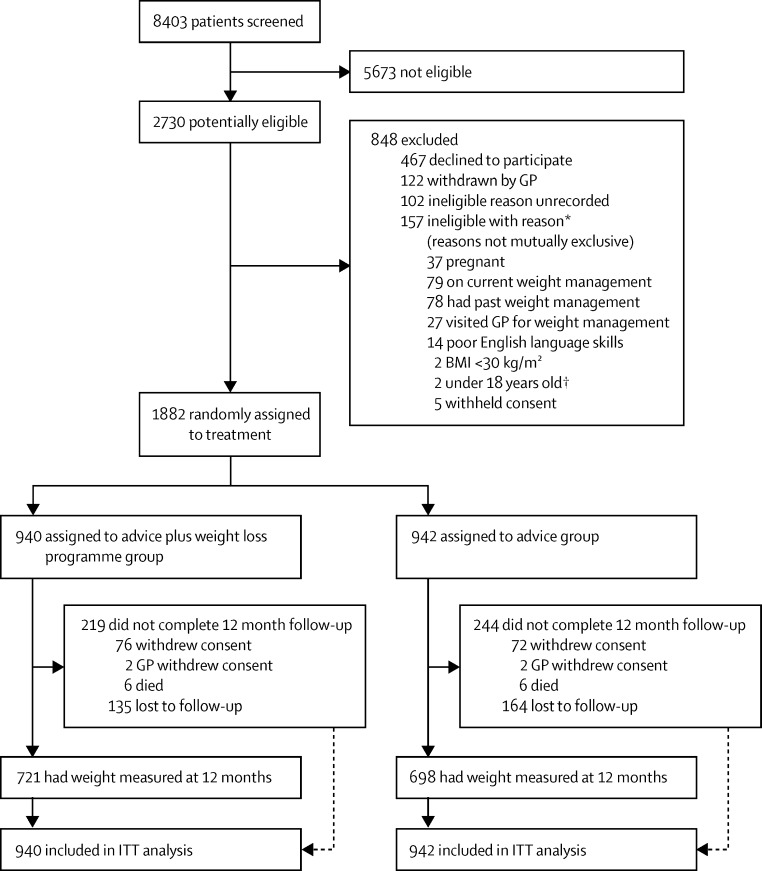
Trial profile GP=general practitioner. BMI=body-mass index. ITT=intention to treat. *Reasons not mutually exclusive. †These patients were mistakenly deemed potentially eligible.

**Table 1:** Baseline characteristics

		**Advice (n=942)**	**Support (n=940)**
Age (years)		56·2 (15·6)	55·8 (16·5)
Gender			
	Male	405 (43·0%)	401 (42·7%)
	Female	537 (57·0%)	539 (57·3%)
Weight (kg)		98·3 (17·6)	97·1 (15·5)
Height (cm)		167·2 (9·6)	166·9 (9·7)
Body-mass index (kg/m^2^)		35·1 (5·1)	34·8 (4·6)
Percentage body fat		40·9% (7·6)	40·4% (7·5)
Socioeconomic status (IMD score)*		15·7 (11·8)	16·4 (12·6)
Ethnic origin			
	White	902 (96%)	884 (94%)
	Black Caribbean	4 (<1%)	10 (1%)
	Black African	7 (1%)	4 (<1%)
	Mixed	0	6 (1%)
	Black Other	1 (<1%)	2 (<1%)
	Chinese	1 (<1%)	2 (<1%)
	Indian	7 (1%)	12 (1%)
	Pakistani	5 (1%)	5 (1%)
	Bangladeshi	4 (<1%)	1 (<1%)
	Other Asian	4 (<1%)	8 (1%)
	Other	7 (1%)	6 (1%)

Data are mean SD or n (%). Ethnic origin was self-defined. IMD=Index of Multiple Deprivation.

* IMD score is an area-based deprivation score, with the English mean being 21·7 (SD 15·6) and higher scores representing greater deprivation.

**Table 2:** Participant ratings of appropriateness and helpfulness of brief intervention

	**Advice**	**Support**
**Appropriateness**		
Patients included in analysis	932	922
Not at all appropriate	4 (<1%)	4 (<1%)
Not appropriate	11 (1%)	11 (1%)
Neither appropriate nor inappropriate	63 (7%)	55 (6%)
Appropriate	364 (39%)	400 (43%)
Very appropriate	490 (53%)	451 (49%)
Adjusted odds ratio (95% CI)*	..	0·89 (0·75–1·07)
p value	..	0·21
**Helpfulness**		
Patients included in analysis	933	922
Not at all helpful	9 (1%)	5 (1%)
Not helpful	12 (1%)	19 (2%)
Neither helpful nor unhelpful	106 (11%)	85 (9%)
Helpful	435 (47%)	442 (48%)
Very helpful	371 (40%)	371 (40%)
Adjusted odds ratio (95% CI)*	..	1·05 (0·89–1·26)
p value	..	0·54

Data are n (%) unless stated otherwise. Patients who did not return to the researcher to complete this assessment were not included in the analysis.

* Ordinal logistic mixed-effects model with fixed effect for randomised group and random effects for physicians.

**Table 3:** Weight loss actions taken by 3 and 12 months

	**Advice**	**Support**
**Actions taken by 3 months**		
Patients included in analysis	520	512
Effective action	56 (11%)	276 (54%)
Self-help action	354 (68%)	165 (32%)
No action	110 (21%)	71 (14%)
Adjusted odds ratio (95% CI)*	..	5·01 (3·86–6·50)
p value	..	<0·0001
**Actions Taken by 12 Months**		
Patients included in analysis	711	682
Effective action	96 (14%)	348 (51%)
Self-help action	436 (61%)	239 (35%)
No action	179 (25%)	95 (14%)
Adjusted odds ratio (95% CI)*	..	4·33 (3·48–5·39)
p value	..	<0·0001

Data are n (%) unless stated otherwise. Data are missing for patients who were not followed up at these points. There was no imputation for missing data. Effective action means a behavioural weight loss programme, medication, or meal replacement programme. Self-help action means reduced energy intake or increased physical activity without professional input.

* Ordinal logistic mixed-effects model with fixed effect for randomised group and random effects for physicians.

**Table 4:** Participants who lost at least 5% and 10% of their bodyweight from baseline to 12 months

	**Advice (n=942)**	**Support (n=940)**
**Lost at least 5% bodyweight**		
Number of patients (%)	131 (14%)	238 (25%)
Adjusted odds ratio (95% CI)*	..	2·11 (1·67–2·68)
p value	..	<0·0001
**Lost at least 10% bodyweight**		
Number of patients (%)	53 (6%)	117 (12%)
Adjusted odds ratio (95% CI)*	..	2·41 (1·72–3·38)
p value	..	<0·0001

* Logistic mixed-effects model with fixed effect for randomised group and random effects for physicians.

## Panel Typical physician intervention

Physician: While you're here, I just wanted to talk about your weight. You know the best way to lose weight is to go to [Slimming World or Rosemary Conley] and that's available free on the NHS?

Patient: Oh?

Physician: Yes, and I can refer you now if you are willing to give that a try?

Patient: Yes, ok.

Physician: Ok, what you need to do is take this envelope back outside to the person who weighed you and they will book you into the weight loss course now.

Patient: Ok.

Physician: Good, but I'd like to see how you're getting on, so come and see me again in 4 weeks, please. Ok?

Patient: Ok, see you then.
