# Supplementary material for: Screening and brief intervention for obesity in primary care: a parallel, two-arm, randomised trial
Source: Lancet. 2016 Nov 19;388(10059):2492–500. doi: 10.1016/S0140-6736(16)31893-1 (PMC5121130; doi:10.1016/S0140-6736(16)31893-1)
Supplement: Supplementary appendix [file mmc1.pdf]

THE LANCET

Supplementary appendix

This appendix formed part of the original submission and has been peer reviewed. We post it as supplied by the authors.

Supplement to: Aveyard P, Lewis A, Tearne S, et al. Screening and brief intervention for obesity in primary care: a parallel, two-arm, randomised trial. *Lancet* 2016; published online Oct 24. http://dx.doi.org/10.1016/S0140-6736(16)31893-1.

Appendix Table 1 Physician fidelity to the intervention protocols

Control intervention*		Active intervention†	
**Stressed the importance of weight loss**	100 (90·9%)	Described support as the most effective way to lose weight	89 (84·0%)
**Provided example/s of health conditions that would be prevented/ameliorated**	99 (90·0%)	Free referral option described	89 (84·0%)
		Offered referral	87 (82·1%)
		Offered the intervention as if coming from the physician	88 (83·0%)
		Asked patient to make an appointment with weight management programme before leaving	77 (72·6%)
		Offered follow-up with physician	55 (51·9%)

* n=110 recordings, † n=106 recordings

Appendix Table 2 sensitivity analyses for the primary outcome of weight loss at 12 months with different imputation methods and none for missing data

	BOCF with randomness*		Last observation carried forward†		Multiple Imputation‡		Completers Only§		Adjustment for predictors of missingness¶	
	Control N=942	Active N=940	Control N=942	Active N=940	Control N=942	Active N=940	Control N=698	Active N=721	Control N=942	Active N=940
**Mean weight change (standard deviation)**	1·05 (5·62)	2·42 (6·58)	1·22 (5·98)	2·64 (6·56)	1·60 (6·64)	2·90 (7·26)	1·41 (6·35)	3·17 (7·25)	1·04 (5·51)	2·43 (6·49)
**Adjusted difference in treatment effect (95%CI)**	-1·41 (-1·96; -0·86)		-1·47 (-2·03; -0·90)		-1·31 (-2·01; -0·62)		-1·80 (-2·50; -1·09)		-1·41 (-1·95; -0·88)	
**p-value**	<0·0001		<0·0001		<0·0001		<0·0001		<0·0001	

All models analysed with linear mixed effects model with covariates for randomised group and baseline weight, and random effects for GP

*** Missing data at 12 months replaced by random variable with weight at baseline plus a randomly generated number with an SD of 2·3, the SD of people whose weight was not missing**

† Missing data at 12 months replaced by self-reported weight at 3 months if available or baseline weight if not

‡ Missing data at 12 months created with multiple imputation using age, height, sex, ethnicity and baseline weight

§ Missing data at 12 months excluded

¶ Additional adjustment for gender, age and IMD score, predictors of missing follow-up data

Brief description of health economic modelling

A fuller description of the modelling and its outputs will be provided in a separate paper. The UKHF model simulated the impact on the population of the UK and the US using data from the Health Survey for England and National Health and Nutrition and Examination Surveys respectively. A BMI growth equation using Monte Carlo techniques simulated the effect of age on the cohort. Every year until death, individuals gained weight and consequently increased BMI in line with projections based on the national survey data. As a consequence of age and increased BMI, we modelled the incidence of BMI-related disease. The model tracked BMI and the incidence, prevalence, costs, QALYs, of BMI-related diseases of individuals over their lifetime, enabling the analysis of cost-effectiveness of the modelled interventions. In the start year of each simulation the individuals in the cohort are randomly assigned a BMI from the distribution observed in the BWeL study and are randomly assigned diseases based on the current prevalence rates in England obtained from the literature. Cumulative incidence, direct costs and ICER are outputs of the microsimulation.

In one scenario, we modelled the prevalence of obesity over 20 years and the impact on health and health service costs assuming no new interventions. In the alternative scenario, we modelled the effect of primary care physicians making one brief intervention each year to the 80% of the population that consulted in that year and were obese. We assumed that the effect of the BWeL intervention remained constant each year. For people who did not consult in any given year, their weight followed the BMI trends in the base model.
